# Identification of FOXK1 and SEMA7A as key genes associated with m^6^A-related programmed cell death in diabetic retinopathy

**DOI:** 10.1186/s41065-026-00710-w

**Published:** 2026-07-10

**Authors:** Jie Yang, Zhenyu Wu, Ningning Dong

**Affiliations:** 1 Zhejiang Hospital, Hangzhou, Zhejiang 310000 China; 2 Eye Hospital of Wenzhou Medical University, Wenzhou, Zhejiang 325000 China

**Keywords:** Diabetic retinopathy, m^6^A modification, Programmed cell death, Key genes, Immune infiltration

## Abstract

**Background:**

Diabetic retinopathy (DR), a serious microvascular complication of diabetes, has a complex pathogenic mechanism that is intricately linked to programmed cell death (PCD) and also to N6-methyladenosine (m^6^A) modification. The objective of this research was to pinpoint crucial genes related to m^6^A-related PCD in DR using transcriptomic data, offering novel targets and a theoretical basis for the pathogenesis of DR.

**Methods:**

In this study, transcriptomic data of DR samples and control samples were obtained from a public database. Meanwhile, m^6^A-and PCD-related genes were retrieved from the literature. Candidate genes were identified via differential expression and correlation analyses. Using constructed protein-protein interaction (PPI) networks, a machine learning algorithms screened for feature genes, which underwent expression validation to determine key genes. A predictive nomogram was subsequently developed and its performance evaluated. Enrichment analysis, along with immune infiltration analysis were carried out. Finally, molecular regulatory networks and molecular docking was performed.

**Results:**

Initially, 3,716 differentially expressed genes between DR and control samples (DRDEGs) were identified. By intersecting DRDEGs with PCD-related genes and m⁶A-related differentially expressed genes, followed by Spearman correlation analysis, 58 candidate genes were identified. Subsequently, Forkhead box K1 (FOXK1) and Semaphorin 7 A (SEMA7A) were identified as key genes through PPI, machine learning, and expression analyses. Furthermore, the two key genes constructed a well accurate nomogram for DR diagnosis. GSEA revealed their critical roles in DR pathogenesis. Moreover, immune infiltration analysis highlighted the involvement of immune dysregulation in DR. The constructed TF-mRNA-miRNA regulatory network contained 2 key genes, 14 transcription factors, and 12 miRNAs (e.g. BRCA1-FOXK1-mmu-miR-7234-3p). Molecular docking showed that decitabine and other drugs bound well to FOXK1,warranting further experimental investigation into their therapeutic efficacy.

**Conclusion:**

This study identified FOXK1 and SEMA7A as key genes in DR related to m^6^A-associated programmed cell death, which may provide a new direction for subsequent research on diagnosis and treatment.

**Supplementary Information:**

The online version contains supplementary material available at https://doi.org/10.1186/s41065-026-00710-w.

## Introduction

As a leading microvascular complication of diabetes, diabetic retinopathy (DR) involves retinal vascular damage caused by chronic hyperglycemia. This damage is a primary cause of visual impairment and blindness in affected individuals [1]. Its pathogenesis involves multiple factors: hyperglycemia drives an imbalance in retinal ceramide metabolism, triggering vascular leakage and ischemia [2]. In addition, inflammatory pathways are upregulated through autocrine and paracrine signaling between human retinal microvascular endothelial cells (hRMECs) and Müller glial cells. This intercellular crosstalk promotes the secretion of angiogenic factors such as IL-8, which in turn drives disease progression [3]. Early clinical manifestations include microaneurysms, hemorrhages, and exudates, while advanced stages are characterized by neovascularization, vitreous hemorrhage, and tractional retinal detachment, which greatly impair patients’ quality of life [1]. Current treatment strategies have significant limitations: Anti-VEGF agents primarily target vascular leakage and neovascularization, yet exhibit inadequate response in a subset of patients; laser photocoagulation destroys retinal tissue and may cause visual field defects; vitrectomy is an invasive surgical procedure with high complication risks [1, 3]. Therefore, exploring novel molecular mechanisms and therapeutic targets is urgently needed.

Programmed cell death (PCD) encompasses apoptosis, autophagy, ferroptosis, pyroptosis, and necroptosis, and its dysregulation is strongly implicated in DR pathogenesis [4]. For instance, excessive apoptosis of retinal ganglion cells and vascular endothelial cells has been documented in DR patients, and suppressing apoptotic pathways protects against retinal ischemia-reperfusion injury [5]. Furthermore, PCD is associated with inflammatory responses and vascular abnormalities in DR, thereby exacerbating disease progression. Pyroptosis promotes the release of the inflammatory cytokine IL-1β by activating the NLRP3 inflammasome, which aggravates retinal vascular leakage and neuroinflammation [6, 7]. N6-methyladenosine (m^6^A), the most common RNA methylation modification, is dynamically regulated by methyltransferases (e.g., METTL3) and demethylases (e.g., ALKBH5). It governs mRNA stability, translation efficiency and splicing [8, 9]. ALKBH5 may delay the progression of DR by reducing ferroptosis through the m^6^A-YTHDF1-ACSL4 axis, thereby providing a new paradigm for the treatment of DR [10]. Additionally, m^6^A modification has been found to influence retinal microvascular endothelial cell function by modulating angiogenesis-related genes [11, 12]. Importantly, studies have shown that in various diseases, such as cancer and cardiovascular disease, there is crosstalk and mutual regulation between m⁶A modification and PCD [13, 14]. However, the crosstalk between m⁶A and PCD in DR remains almost unexplored.

To fill this gap, the present study leveraged transcriptomic data from public databases to systematically identify key genes associated with m⁶A-related PCD in DR. We applied differential expression analysis, protein-protein interaction networks, machine learning algorithms, immune infiltration analysis, and molecular docking. Our goal is to provide new biomarkers and potential therapeutic targets for DR, and to offer a theoretical basis for precision treatment.

## Method and materials

### Data collection

The diabetic retinopathy (DR) - related datasets (GSE221521, GSE185011 and GSE248284) were retrieved from the Gene Expression Omnibus (GEO) repository (https://www.ncbi.nlm.nih.gov/geo/). The GSE221521 dataset (GPL24676) comprised whole-blood transcriptomic profiles from 69 DR specimens and 50 control specimens as training set. GSE185011 (GPL24676) [15] comprised 5 DR specimens and 5 control specimens as validation set. Single-cell dataset, GSE248284 (GPL24676) was sourced from the GEO repository, comprising 3 DR samples and 3 healthy control samples. Meanwhile, Totally 27 N6-methyladenosine modification (m^6^A)-related genes (m^6^A-RGs) were obtained from the literature (Supplementary Table 1) [16]. Meanwhile, 1,548 PCD-related genes (PCDRGs) were retrieved from the literature (Supplementary Table 2) [17]. Notably, although these gene sets are derived from non-DR diseases, m^6^A modification and PCD pathways are highly conserved in metabolic diseases (e.g., diabetes, cardiovascular diseases) [14, 18–20], and existing studies have suggested that they share key regulatory nodes among various microvascular complications [21, 22].

### Differential expression analysis

Differential gene expression analysis between DR and control samples in the training dataset was conducted through the DESeq2 R package (version 1.38.0) [23] to extract differentially expressed genes, termed DRDEGs. Statistical thresholds for significant differential expression included p-value < 0.05 combined with absolute Log2 fold-change values exceeding 0.5. Visual representation of these findings was achieved by generating volcano plots via the ggplot2 R package (version 3.5.1) [24], highlighting the most significantly upregulated (top 10) and downregulated (bottom 10) transcripts for enhanced interpretation.

Furthermore, to investigate the functional significance of N6-methyladenosine-related genes (m^6^A-RGs) within diabetic retinopathy pathogenesis, we implemented single-sample Gene Set Enrichment Analysis (ssGSEA) through the GSVA R computational package (version 1.46.0) [25]. This analytical approach enabled the assessment of pathway activity scores across individual samples, providing insights into the biological processes associated with m^6^A modification in DR development. The ssGSEA algorithm calculated enrichment scores for each sample in the training dataset based on m^6^A-RGs. Samples were then stratified into high- and low-score groups according to the median of the ssGSEA scores. And statistical comparisons of enrichment scores between these groups were performed, with statistical significance determined at *p* < 0.05.

Subsequently, the R package “DESeq2” (v 1.38.0) was employed to identify DEGs between the high- and low-score groups, and significant thresholds set as |Log_2_FC| > 0.5 and *p* < 0.05 were considered significant. These identified DEGs were considered as m^6^ADEGs. For visualization, the R package “ggplot2” (v 3.5.1) was used to construct a volcano plot illustrating to demonstrate the distribution of m^6^ADEGs and highlight the 10 genes with the highest up-regulated and 10 genes with the lowest down-regulated genes. Notably, this approach assumed that the expression of m⁶A regulatory genes could represent the global m⁶A modification level. However, this assumption lacked experimental verification based on the current dataset, and the obtained genes were correlated with the expression pattern of m⁶A regulators rather than proven targets directly affected by m⁶A modification levels.

### Intersection and correlation analysis

Furthermore, the intersection of DRDEGs and PCDRGs was determined to identify differentially expressed genes related to PCD. Similarly, the intersection DRDEGs and the m^6^ADEGs was determined. The R package “ggvenn” (v 0.1.9) [26] was employed to visualize the intersections.

To further refine related genes associated with both m^6^A related-PCD, Spearman correlation analysis was performed on the two intersecting genes using the R package “psych” (v 2.2.9) [27]. We calculated all pairwise correlation coefficients between the two sets of intersecting genes. Only gene pairs with an absolute correlation coefficient greater than 0.8 and a p-value less than 0.05 were retained. This threshold is commonly used to identify strong co-expression relationships and reduce false positives in multi-step bioinformatics pipelines. Importantly, all gene pairs meeting this criterion were included in the analysis. After removing duplicates, the unique genes were designated as candidate genes for subsequent analyses. Meanwhile, we performed sensitivity analyses using gradient thresholds of |cor| > 0.5, 0.6, 0.7 and 0.8.

### Enrichment analysis of candidate genes

To elucidate the biological significance and disease relevance of identified candidate genes, we performed comprehensive functional annotation through three complementary enrichment approaches: Gene Ontology (GO) for biological processes characterization, Kyoto Encyclopedia of Genes and Genomes (KEGG) for metabolic and signaling pathway analysis, and Disease Ontology (DO) for pathological condition mapping. These analyses were executed via the clusterProfiler R computational framework (version 4.2.2) [28] in conjunction with the human genome annotation database “org.Hs.eg.db”. Statistical significance was determined using a threshold of p-value less than 0.05, ensuring robust identification of biologically meaningful associations. Specifically, GO enrichment analysis categorized genes into three functional domains: biological process (BP), cellular component (CC), and molecular function (MF), while KEGG analysis identified significant biological pathways, and DO enrichment analysis identified the potential associations between candidate genes and known diseases. The top 5 pathways or diseases were performed according to the rank by the the significance value from high to low.

### Protein-protein interaction (PPI) network construction and machine learning algorithm for feature genes

Candidate genes were submitted to the Search Tool for the Retrieval of Interacting Genes/Proteins (STRING) database platform (https://string-db.org/) to assess the interactions between proteins encoded by candidate genes using a confidence threshold > 0.4. Following acquisition of interaction data, solitary nodes were excluded. Cytoscape (v 3.7.2) [29] was subsequently employed to construct the PPI network. Network genes were designated as hub genes for subsequent analysis. To additionally filter DR-associated genes from hub genes, the R package “glmnet” (v 4.1.4) was employed to execute least absolute shrinkage and selection operator (LASSO) regression analysis. Optimal lambda parameters were established when 10-fold cross-validation error reached minimum. Subsequently, hub genes were designated as feature genes upon optimal lambda determination.

### Key genes screening

Within the training and validation cohorts, the R package “DESeq2” (v 1.38.0) was employed to compute variations in feature gene expression levels among DR specimens and control specimens. Genes exhibiting uniform expression patterns across both cohorts and notable variations among DR specimens and control specimens were identified as critical genes (*p* < 0.05).

### Nomogram construction and evaluation

A nomogram for predicting DR probability was developed using the R package “rms” (v 6.5.0) (https://cran.r-project.org/package=rms), incorporating expression levels of key genes derived from the training dataset.

In order to appraise the predictive accuracy of the nomogram, the R package “ResourceSelection” (v 0.3.5) (https://cran.r-project.org/package=ResourceSelection) was employed to plot calibration curves. Specifically, the closer the slope of the calibration curve approaches 1, the better the predictive efficacy of the nomogram.

Furthermore, we assessed the clinical applicability of the constructed nomogram through decision curve analysis (DCA) incorporating the identified key genes. The predictive performance of the nomogram for DR within the training cohort was determined by generating receiver operating characteristic (ROC) curves utilizing the “pROC” R package (version 1.18.0). Additionally, the practical clinical implementation potential of the nomogram was examined by developing clinical impact curves through the “rmda” R package (version 1.6) (https://github.com/mdbrown/rmda).

### Chromosome and subcellular localization analyses

For chromosomal positioning analysis, the R package “OmicCircos” (v 1.36.0) was utilized using critical genes in the training cohort. Circular visualization was generated to display the chromosomal distribution of critical genes.

Genomic sequences of critical genes were retrieved from the National Center for Biotechnology Information (NCBI) repository (https://www.ncbi.nlm.nih.gov/), for subsequent subcellular positioning analysis. These sequences were subsequently submitted to the mRNALocater platform (http://bio-bigdata.cn/mRNALocater/) to obtain 5 subcellular positioning prediction values for each critical gene. The position with the highest value was established as the ultimate subcellular positioning.

### Gene Set Enrichment Analysis (GSEA) of key genes

To elucidate the underlying molecular mechanisms and regulatory pathways associated with pivotal genes identified within our training dataset, Gene Set Enrichment Analysis (GSEA) was implemented as the analytical framework. As a preliminary step in this computational approach, we computed Spearman’s rank correlation coefficients by assessing the relationship between individual key genes and the comprehensive transcriptomic expression matrix, thereby establishing quantitative correlation metrics for subsequent pathway enrichment evaluation. These genes were subsequently ordered from highest to lowest correlation coefficients. The GSEA procedure was then implemented via the “clusterProfiler” R package (version 4.2.2) [28], employing the “c2.cp.v7.2.symbols.gmt” gene collection obtained from MSigDB (https://www.gsea-msigdb.org/gsea/msigdb). Statistical significance for enrichment was determined using criteria of |NES| > 1 and adjusted p-value < 0.05.

### Immune infiltration analysis

To characterize immunological profile variations between diabetic retinopathy cases and healthy control subjects, we implemented single-sample Gene Set Enrichment Analysis (ssGSEA) methodology for quantifying the relative enrichment scores of twenty-eight distinctive immune cell type signatures [30] across our study population. This computational analysis was executed through the Gene Set Variation Analysis (GSVA) R computational package (version 1.46.0), enabling systematic evaluation of immune cell infiltration patterns within the training dataset. Cases exhibiting p-values exceeding 0.05 were filtered out from subsequent analyses. Following this, we applied the Wilcoxon rank-sum test to evaluate differential immune cell infiltration patterns between DR and control groups, establishing statistical significance at *p* < 0.05.Additionally, the Spearman correlation coefficients (|cor|>0.3, *P* < 0.05) between differential immune cells and between key genes and differential immune cells were calculated using the R package “psych” to explore their associations.

### Processing of the scRNA-seq data

Single-cell analyses for GSE248284 were performed in Seurat (v 5.0.1). Quality control filters were: 200 < nFeature_RNA < 5,000; percent.mt < 20%; 200 < nCount_RNA < 20,000. Data were normalized and scaled with NormalizeData and ScaleData. Highly variable genes (HVGs; *n* = 2,000) were identified using the vst method (FindVariableFeatures); the top 10 HVGs were labeled (LabelPoints). Principal component analysis (RunPCA) was followed by ElbowPlot and JackStraw to select significant PCs (*P* < 0.05). Cells were clustered (resolution = 0.3) and embedded by UMAP. Next, cell type annotation was performed through marker genes from published literature [31]. In addition, bar charts were used to display the proportions of different cell types in the DR group and the control group. Finally, bubble plot was employed to visualize the expression of key genes across different cell types.

### Molecular regulatory and drug network construction and molecular docking

To predict the transcription factors (TFs) that target and regulate key genes, the Jaspar database (https://jaspar.genereg.net/) were utilized. To predict the microRNAs (miRNAs) that target and regulate key genes, the targetscan (https://www.targetscan.org/) and MicroRNA Target Prediction and Functional Annotations Database (miRDB) databases (http://www.mirdb.org) were used. The intersection of the miRNAs predicted by both databases were identified as Int-miRNAs. The Int-miRNAs were used to the construction of the miRNA-mRNA networks. Finally, the Cytoscape (v 3.7.2) was utilized to construct a TF-mRNA-miRNA merge network.

We sought to identify candidate therapeutic compounds that could target the identified critical genes by utilizing the Drug Signatures Database (DSigDB) (https://dsigdb.tanlab.org/DSigDBv1.0/) for drug prediction and quantification of drug-gene interaction coefficients. Subsequently, we employed Cytoscape software (version 3.7.2) to visualize and analyze the molecular interaction networks formed between the predicted compounds and the protein products of these critical genes.

Furthermore, the predicted drugs were selected to perform the molecular docking analysis. Three-dimensional structural models of therapeutic compounds were retrieved from the PubChem database (https://pubchem.ncbi.nlm.nih.gov/) [32], while crystallographic conformations of protein products corresponding to critical genes were obtained from the UniProt database (https://www.uniprot.org/) [33]. The docking was completed using AutoDock Vina software (v 1.2.5) [34], with a threshold set at binding energy < -5.0 kcal/mol, and then visualized using PyMOL software (v 2.6.0) [35].

### Reverse transcription-quantitative polymerase chain reaction (RT-qPCR) experiment

This study collected 10 fresh human peripheral blood samples (5 controls and 5 DR cases) from the clinic in the Zhejiang hospital, which were completely independent of the GSE185011 dataset. Clinical characteristics of the participants are summarized in Supplementary Table 3. The study had the approval of the ZJHIRB-019 K ethics committee. Total RNA was extracted using a modified TRIzol-based protocol, followed by cDNA synthesis via reverse transcription. The expression levels of target genes and the reference gene were quantified by RT-qPCR. Primer specificity was verified via melting curve analysis, with single-peak profiles indicating specific amplification. The experimental methods and primer sequences (Supplementary Table 4). Quality assessment of the extracted RNA showed that A260/A230 ratios were below 0.4 across all samples, suggesting potential residual contaminants. We acknowledge that the unsatisfactory RNA purity reflected by low A260/A230 ratios is a limitation of this pilot study. Future experiments will employ column-based purification or additional wash steps to improve RNA purity. Relative gene expression was calculated using the 2^–△△Ct^ method, and statistical analysis was performed with GraphPad Prism (v 10.1.2) [36], setting a significance threshold of *P* < 0.05.

### Statistical analysis

We conducted all bioinformatics analyses using R software (version 4.2.2). Wilcoxon rank-sum tests were applied to assess differential patterns between DR cases and control subjects. Spearman’s rank correlation was performed to examine associations between gene expression and immune cell profiles. Statistical significance was defined as *p* < 0.05 across all analyses.

## Results

### Identification of DEGs related to DR and m^6^A

**Fig. 1:**
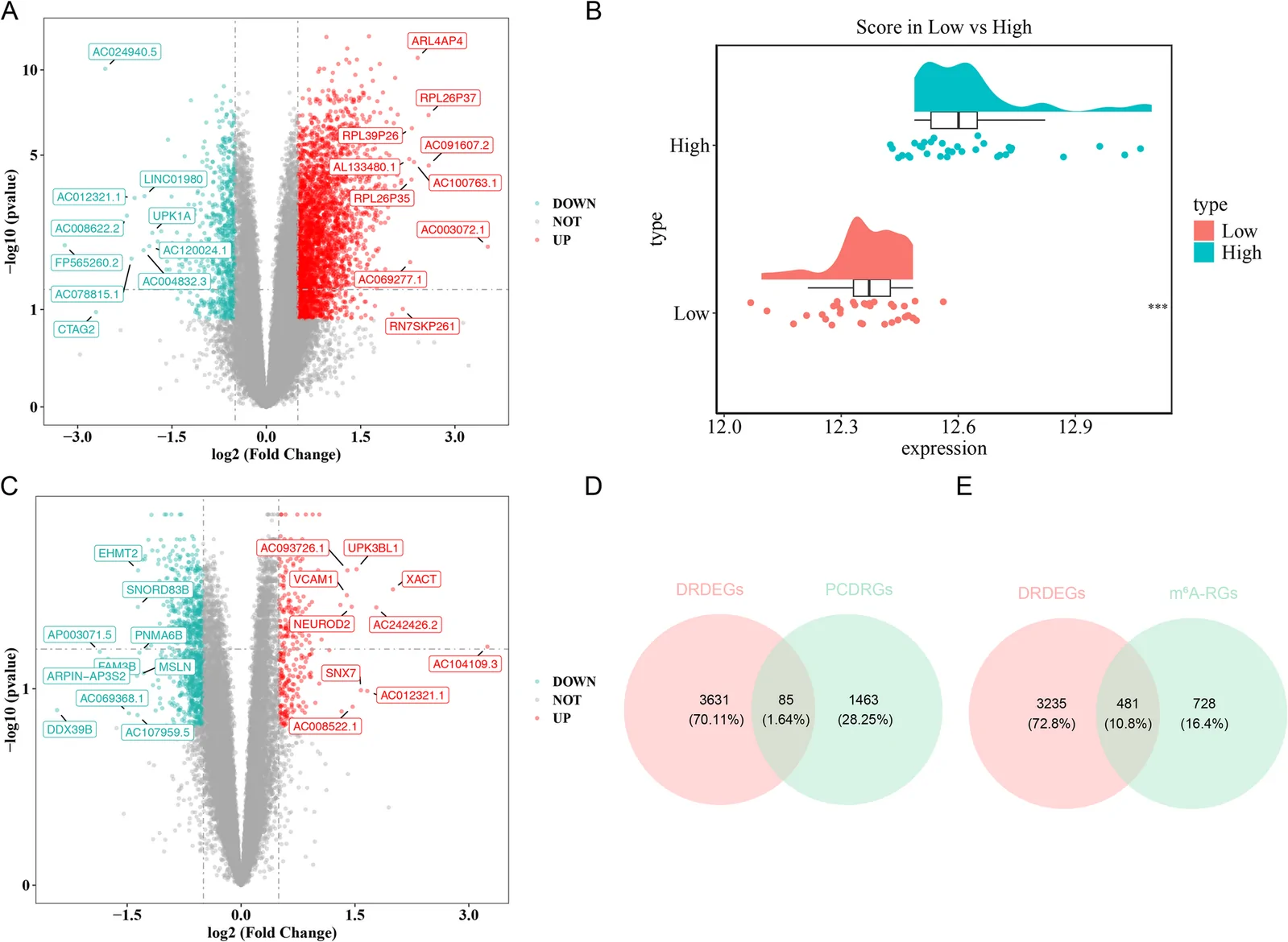
Differential expression analysis and acquisition of intersecting genes. **A** Volcano plot showing the identification of 3,716 differentially expressed genes in DR samples compared to controls. **B** Box plot comparing the ssGSEA enrichment scores of m6A-related gene sets between high- and low-score groups in the training dataset. **C** Volcano plot displaying the 1,209 m^6^A-related differentially expressed genes (m^6^ADEGs) identified between the high- and low-score groups. **D** Venn diagram illustrating the intersection of 3,716 DRDEGs and 1,548 programmed cell death-related genes (PCDRGs), yielding 85 overlapping genes. E: Venn diagram showing the intersection of 3,716 DRDEGs and 1,209 m^6^A-related genes (m^6^A-RGs), identifying 481 overlapping genes

Firstly, a total of 3,716 DRDEGs were identified between DR samples and control samples in the training dataset (*p* < 0.05, |Log_2_FC| > 0.5), comprising 2,940 upregulated and 776 downregulated genes (Fig. 1A and Supplementary Table 5). The significant gene expression changes suggested widespread molecular dysregulation in DR.

Subsequently, to identify more genes which were related to m^6^A, ssGSEA and differential expression analyses were conducted. ssGSEA was first applied to convert expression data of 27 m^6^A-RGs into enrichment scores for each sample within the training cohort. Following the enrichment score calculation, we dichotomized the sample cohort into two distinct groups using the median value as the cutoff threshold: specimens exhibiting above-median scores were classified as high-enrichment, while those below the median were designated as low-enrichment categories. To assess the statistical robustness of observed differences between these binary classifications, we applied the Wilcoxon rank-sum test with significance determined at p-value < 0.05 (Fig. 1B). Afterwards, to further explore m^6^A-related transcriptional changes, differential expression analysis was conducted between the two groups, identifying 1,209 m^6^ADEGs (*p* < 0.05, |Log_2_FC| > 0.05). Among these, 295 m^6^ADEGs were upregulated, while 914 were downregulated (Supplementary Table 6) (Fig. 1C). These results expand the m^6^A-RGs profile to a total of 1,209 m^6^ADEGs, providing a broader perspective on involvement of m^6^A-RGs in DR progression.

### Identification of candidate genes

The intersection of 3,716 DRDEGs and 1,548 PCDRGs was performed, yielding 85 intersecting genes (Fig. 1D). Similarly, the intersection of 3,716 DRDEGs and 1,209 m^6^A-RGs identified 481 intersecting genes (Fig. 1E).

To further screen the genes related to m^6^A and PCD in DR, Spearman correlation analysis was conducted between the 85 intersecting genes and the 481 intersecting genes. As a result, 58 genes including FOXK1 and SEMA7A were identified as candidate genes for further analysis (|cor| > 0.8, *p* < 0.05) (Supplementary Table 7). Sensitivity analysis showed that FOXK1 and SEMA7A were consistently retained as candidate genes across all thresholds, confirming that the identification of these two key genes is not sensitive to the specific cutoff chosen (Supplementary Table 8).

### Enrichment analysis of candidate genes in DR

**Fig. 2:**
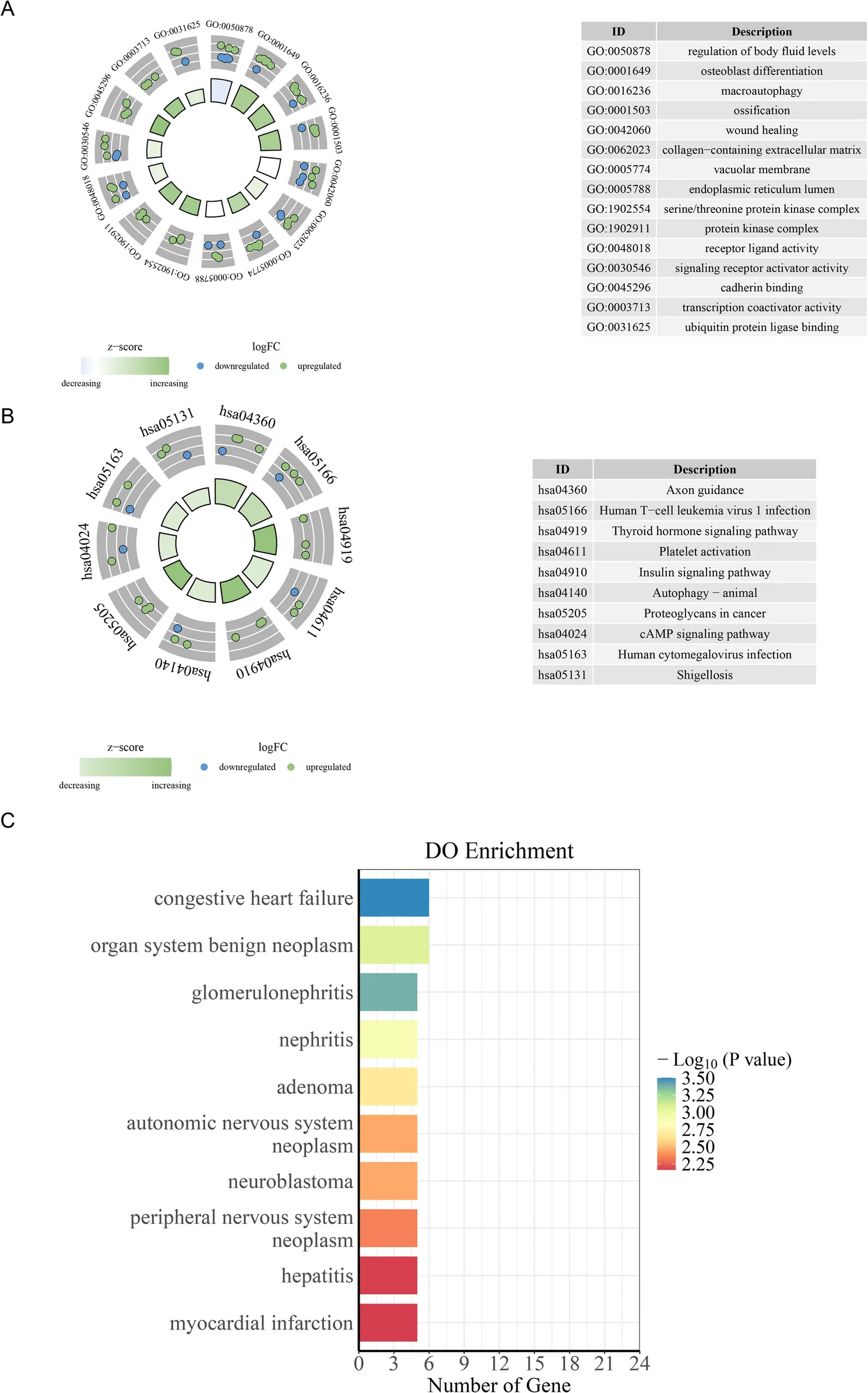
Functional enrichment analysis of candidate genes. **A** GO enrichment analysis of candidate genes. **B** KEGG pathway enrichment analysis of candidate genes. **C** Disease Ontology enrichment analysis

A total of 1,201 significantly enriched GO terms (*p* < 0.05) were identified, including 1,076 in BP, 59 in CC, and 66 in MF. The top 5, ranked according to the gene counts from high to low, BP terms included “regulation of body fluid levels”, “osteoblast differentiation”, “macroautophagy”, “ossification”, and “wound healing”. In the CC category, it was observed that there was significant enrichment in components such as “vacuolar membrane”, “endoplasmic reticulum lumen”, and “serine/threonine protein kinase complex”. The MF included, “receptor-ligand activity”, “transcription coactivator activity”, and “ubiquitin protein ligase binding” (Fig. 2A and Supplementary Table 9).

Meanwhile, 29 KEGG pathways were significantly enriched (*p* < 0.05), with the top pathways, ranked according to the gene counts from high to low, such as “axon guidance”, “platelet activation”, and “insulin signaling pathway.” The analysis revealed that the identified genes participated in neural regulation, immune responses, metabolic control, and platelet function, which are relevant to DR pathogenesis. (Fig. 2B and Supplementary Table 10).

Finally, a total of 219 diseases were significantly enriched (*p* < 0.05), with the top associations, ranked according to the gene counts from high to low, including “adrenal adenoma”, “adult T-cell leukemia/lymphoma”, “bicuspid aortic valve disease”, “congenital diaphragmatic hernia”, and “congestive heart failure.” (Fig. 2C and Supplementary Table 11).

Overall, these findings highlighted the potential roles of the candidate genes in autophagy, immune responses, and systemic disease processes, further supporting their significance in DR pathology and potential as therapeutic targets.

### Identification of key genes in DR

**Fig. 3:**
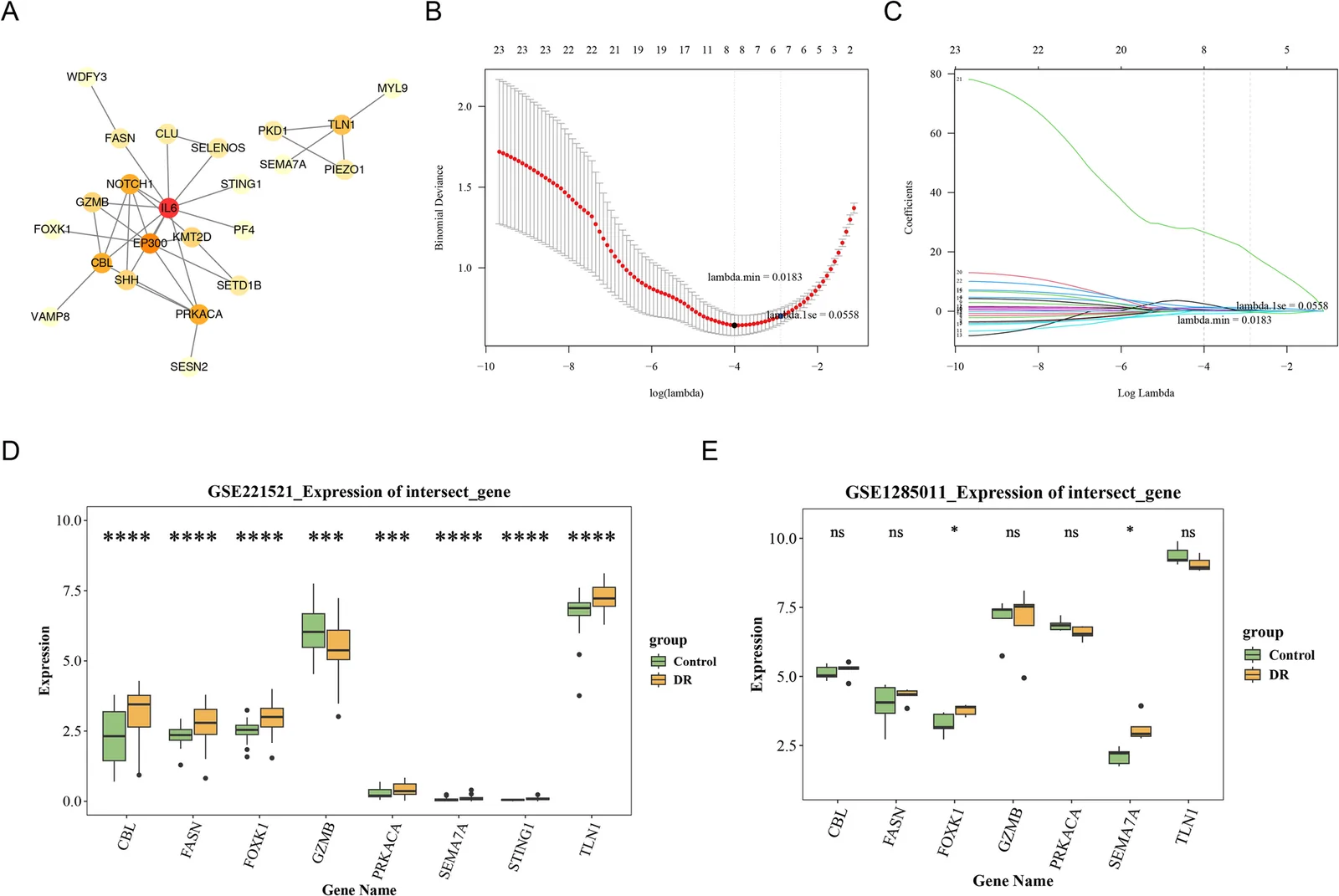
Screening of key genes. **A** Protein-protein interaction (PPI) network of the 58 candidate genes. Nodes with low connectivity were filtered out, revealing 23 hub genes with 33 interaction pairs. IL6 is highlighted as the node with the most connections. **B** LASSO coefficient profiles of the hub genes in the training dataset. **C** Ten-fold cross-validation for tuning parameter (lambda) selection in the LASSO model. The vertical line indicates the optimal lambda value (0.0183) where the model achieved the minimum error, resulting in the selection of eight feature genes. **D** Expression levels of the eight LASSO-selected feature genes (Cbl proto-oncogene (CBL), Fatty acid synthase (FASN), Granzyme B (GZMB), Protein kinase A catalytic subunit alpha (PRKACA), Semaphorin 7 A (SEMA7A), Forkhead box K1 (FOXK1), Stimulator of interferon genes 1 (STING1), and Talin 1 (TLN1) ) in DR versus control samples from the training dataset. **E** Validation of the expression trends for the eight feature genes in an independent validation dataset, comparing DR and control samples

To further screen the candidate genes, a PPI network was constructed with the lower connectivity were excluded, revealing 23 hub genes with 33 interaction pairs (Fig. 3A). As a result, IL6 had the most target genes, such as NOTCH1, EP300, PRKACA. These hub genes were considered for further analysis.

LASSO algorithm was applied to the training dataset to refine the hub genes in DR. The optimal lambda value of 0.0183, selected based on the minimum ten fold cross validation error, resulted in the identification of eight feature genes: Cbl proto-oncogene (CBL), Fatty acid synthase (FASN), Granzyme B (GZMB), Protein kinase A catalytic subunit alpha (PRKACA), Semaphorin 7 A (SEMA7A), Forkhead box K1 (FOXK1), Stimulator of interferon genes 1 (STING1), and Talin 1 (TLN1) (Fig. 3B, C). These genes represented potential research candidates for further functional investigation.

To further validate feature gene expression trends, differential analysis was performed between DR samples and control samples based on the training and validation datasets. FOXK1 and SEMA7A showed a consistent and significant upregulation in DR samples compared to control samples in both datasets (*p* < 0.05) (Fig. 3D, E). As a result, these two feature genes were identified as key genes potentially associated with m^6^A-related PCD in DR. They may serve as downstream effector molecules linking m^6^A modification and PCD, whereas direct regulatory interactions between m^6^A and the two genes remain to be verified via subsequent epitranscriptomic experiments such as MeRIP-qPCR or m^6^A sequencing.

### Evaluation of key gene-based nomogram for DR prediction

**Fig. 4:**
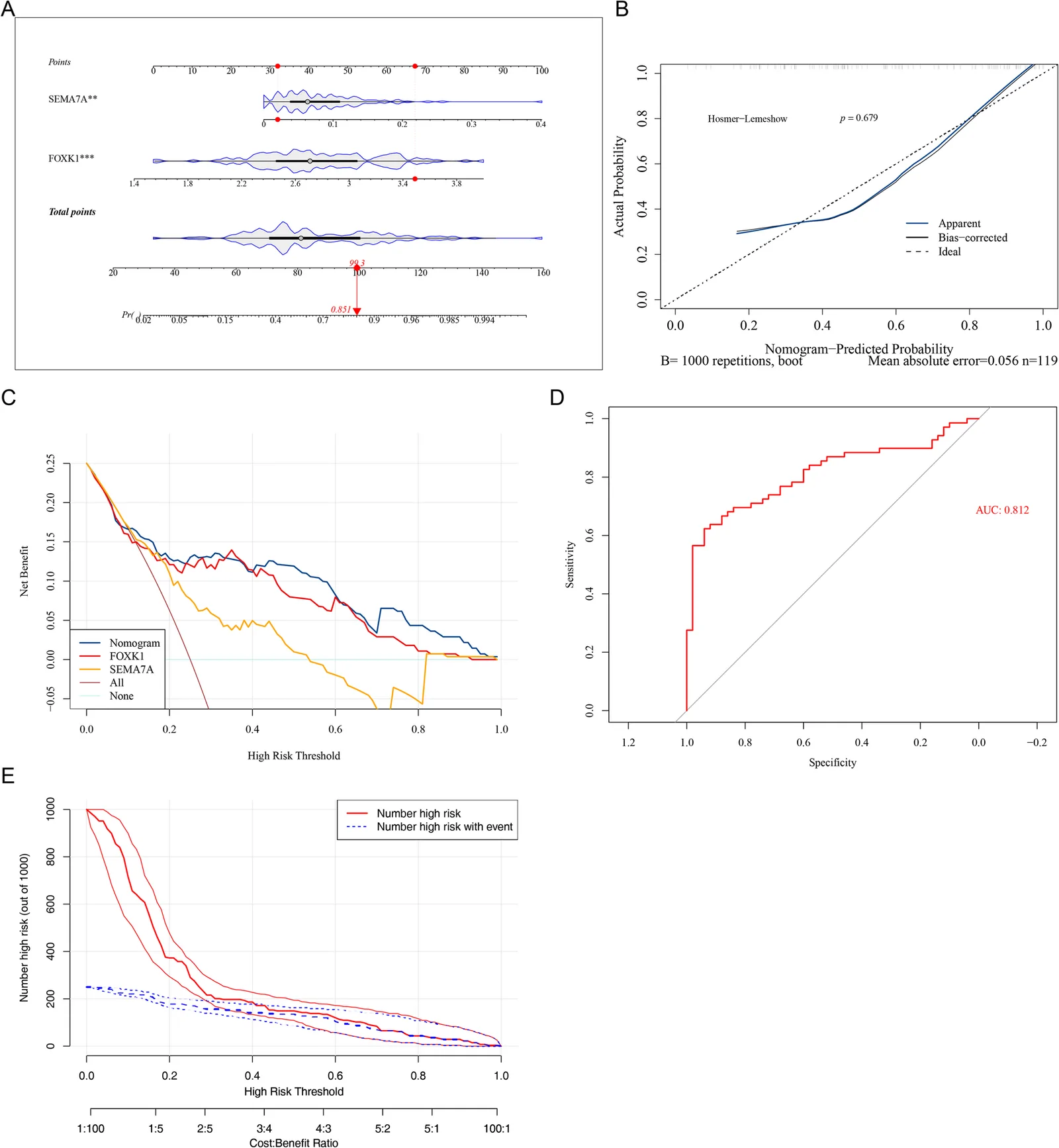
Construction of nomogram. **A** Nomogram constructed using the key genes FOXK1 and SEMA7A to predict the probability of DR onset. The total score was calculated by summing the tick values of all key genes, with higher total scores indicating a greater risk of developing DR. The length of each line segment reflected the magnitude of that gene’s contribution to the outcome event. The “Points” in the figure represented the individual score assigned to each gene based on its specific value, while the “Total Points” denoted the sum of all individual scores. To obtain the predicted probability, a vertical line was drawn downward from the total score along the “Total Points” axis; its intersection with the “Pr (DR)” axis gave the patient’s predicted probability of developing DR. **B** Calibration curve of the nomogram. The solid line represents the observed outcomes against the predicted probabilities. The dashed line (ideal reference) indicates perfect prediction. The Hosmer-Lemeshow (HL) test p-value and Mean Absolute Error (MAE) are shown. **C** Decision curve analysis (DCA) for the nomogram model and the individual key genes, showing the net benefit across different threshold probabilities. **D** Receiver operating characteristic (ROC) curve for the nomogram modele. **E** Clinical impact curve (CIC) analysis for the nomogram, showing the number of subjects classified as high risk and the number of true positives among them across different probability thresholds

A nomogram integrating key genes was developed to predict the probability of DR onset. In this model, the length of each line segment in the nomogram reflected the relative contribution of each gene to the overall risk prediction. Notably, FOXK1 and SEMA7A both had high contribution to the predictive value of the nomogram (Fig. 4A).

As shown in the calibration curves, the slope of the calibration curve was nearly 1, indicating that the nomogram had a highly predictive value. Additionally, the HL test yielded *p* = 0.679, confirming a good model fit. Moreover, the MAE was 0.056, indicating low deviation between predicted and actual DR risks (Fig. 4B). The DCA demonstrated that the predictions according to the nomogram achieved a higher net benefit than those derived from FOXK1 or SEMA7A expression levels (Fig. 4C). The ROC curve showed an AUC of 0.812, indicating favorable predictive performance (Fig. 4D). Additionally, clinical impact curve analysis corroborated the model’s reliability. When examining thresholds above 0.4, the curves representing “Number high risk” and “Number high risk with event” demonstrated remarkable consistency, supporting the model’s effectiveness in identifying high-risk cases (Fig. 4E).

### Chromosomal and subcellular localization of key genes

**Fig. 5:**
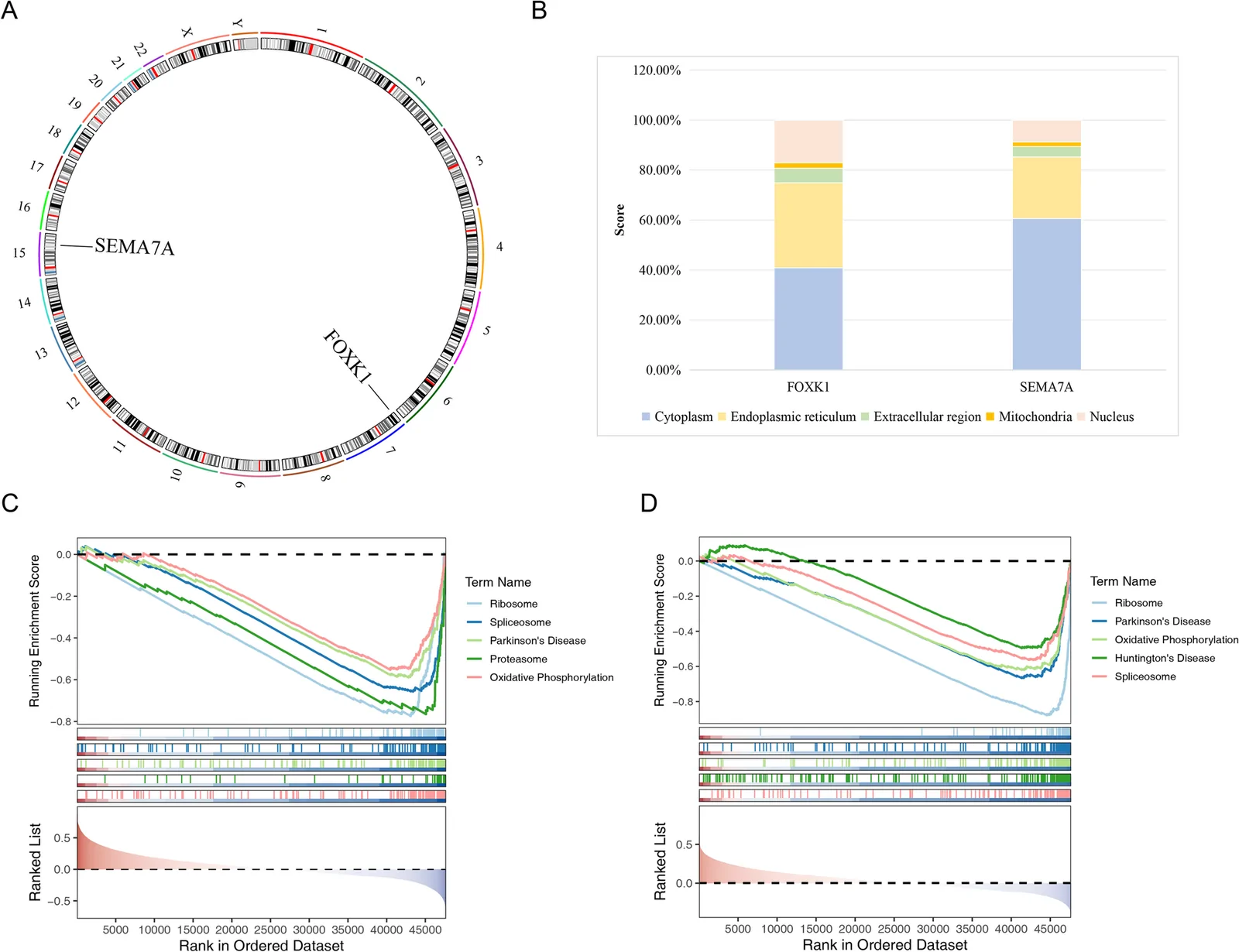
Chromosomal localization, subcellular localization and functional enrichment analysis of key genes. **A** Chromosomal localization analysis. **B** Subcellular localization analysis. **C** Gene Set Enrichment Analysis (GSEA) results for FOXK1 in the training dataset. **D** Gene Set Enrichment Analysis (GSEA) results for SEMA7A in the training dataset

Genome-wide localization analysis revealed that FOXK1 was mapped to chromosome 7, while SEMA7A was located on chromosome 15, as illustrated in the Circos plot (Fig. 5A). These chromosomal positions provided insights into the genomic context of these genes and their potential regulatory elements.

To further investigate their subcellular distribution, sequence-based prediction was conducted. The highest localization scores indicated that both FOXK1 and SEMA7A predominantly reside in the cytoplasm (Fig. 5B). This suggested that they may exert their functional roles primarily in cytoplasmic processes. Further experimental validation was required to elucidate their precise roles in disease progression.

### Biological pathways enriched by key genes

In the training set, GSEA was carried out to identify the biological functions that these two key genes FOXK1 and SEMA7A involved in. Both genes were enriched in “ribosome”, “spliceosome”, and “oxidative phosphorylation”, alongside disease-associated pathways such as “Parkinson’ s disease”. FOXK1 uniquely enriched “proteasome” processes, while SEMA7A showed specific associations with “Huntington’ s disease”. (Fig. 5C, D and Supplementary Tables 12–13). These results indicate that FOXK1 and SEMA7A are statistically associated with biological processes related to protein synthesis and RNA splicing. We speculate that the two genes may regulate protein synthesis and energy metabolism linked to apoptosis in diabetic retinopathy, which needs to be verified by further experiments.

### Immune cell infiltration in DR

**Fig. 6:**
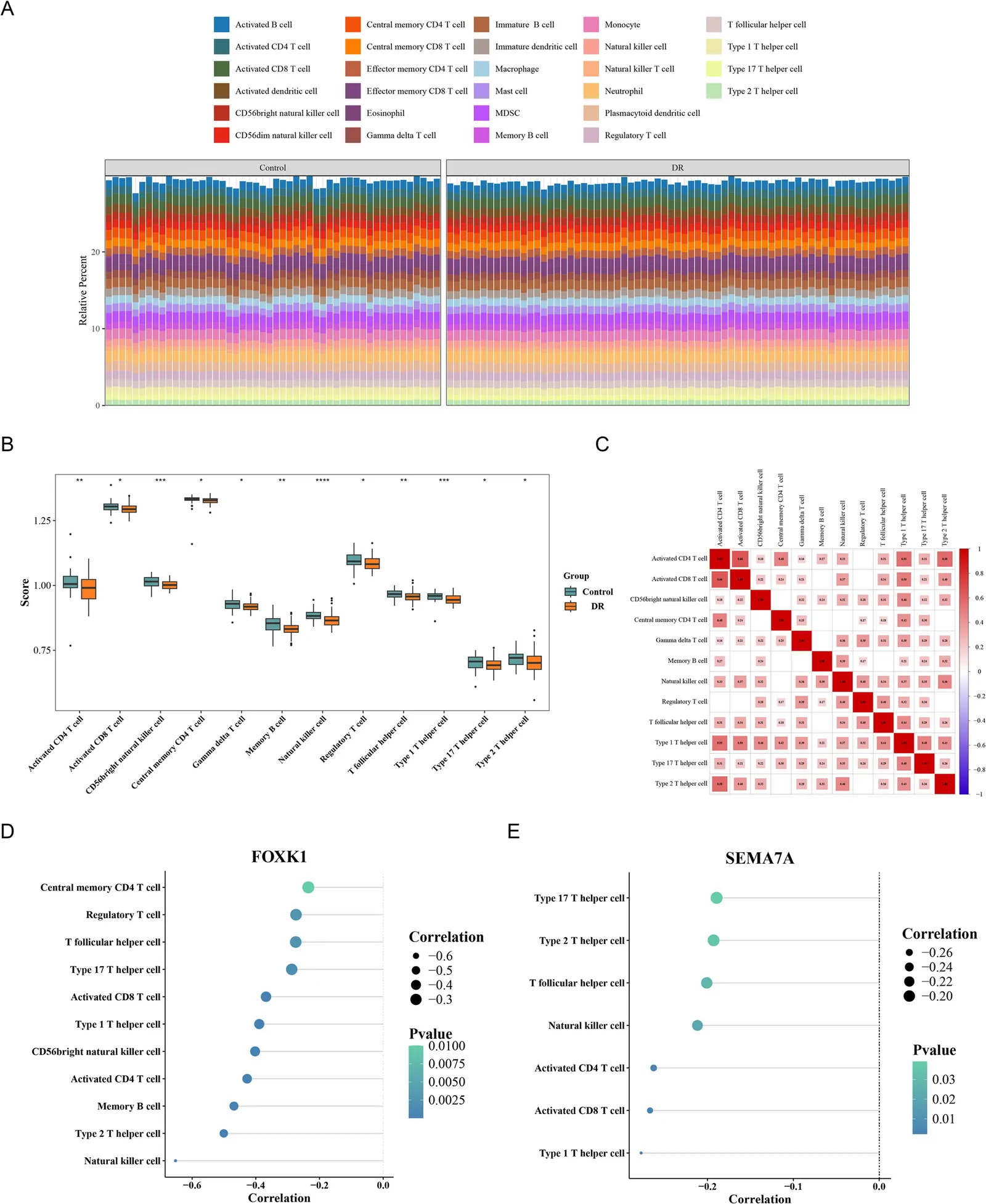
Immune infiltration analysis. **A** Heatmap displaying the abundance of 28 immune cell types in DR and control samples. **B** Box plots comparing the infiltration levels of 12 significantly different immune cell types between DR and control groups. (*p < 0.05, **p < 0.01, ***p < 0.001, ****p < 0.0001). **C** Correlation heatmap showing the associations among the differentially infiltrated immune cell types. **D** Correlation analysis between differentially expressed immune cells and FOXK1. **E** Correlation analysis between differentially expressed immune cells and SEMA7A

To investigate the differences in immune characteristics between DR and control samples, ssGSEA of 28 immune cell types was performed, and immune cell abundance was compared between the two groups (Fig. 6A). Totally, 12 immune cell types exhibited significant differences in infiltration levels (*p* < 0.05), including activated B cell, memory B cell, CD4^+^ T cell, and monocyte (Fig. 6B), highlighting potential immune dysregulation in DR.

Moreover, Spearman correlation analysis was conducted to explore the associations among differentially infiltrated immune cells and their relationships with key genes. Significant positive correlations were observed among the differentially infiltrated immune cells (|cor| > 0.3, *p* < 0.05) (Fig. 6C). Moreover, FOXK1 was significantly negatively correlated with 9 immune cells, specially natural killer cells (cor = -0.65, *p* < 9.54 × 10^− 16^) (Fig. 6D), indicating a potential regulatory role of FOXK1 in immune responses. However, SEMA7A was no significantly to all immune cells in this analysis (Fig. 6E and Supplementary Table 14). These findings indicated that immune cell infiltration was dysregulated in DR, and FOXK1 might played a role in modulating the immune dysregulation, potentially affecting the development and progression of DR. However, it should be noted that these findings represent peripheral blood immune profiles; extrapolation to retinal immune status should be made with caution.

### A comprehensive single-cell sequencing analysis in DR

**Fig. 7:**
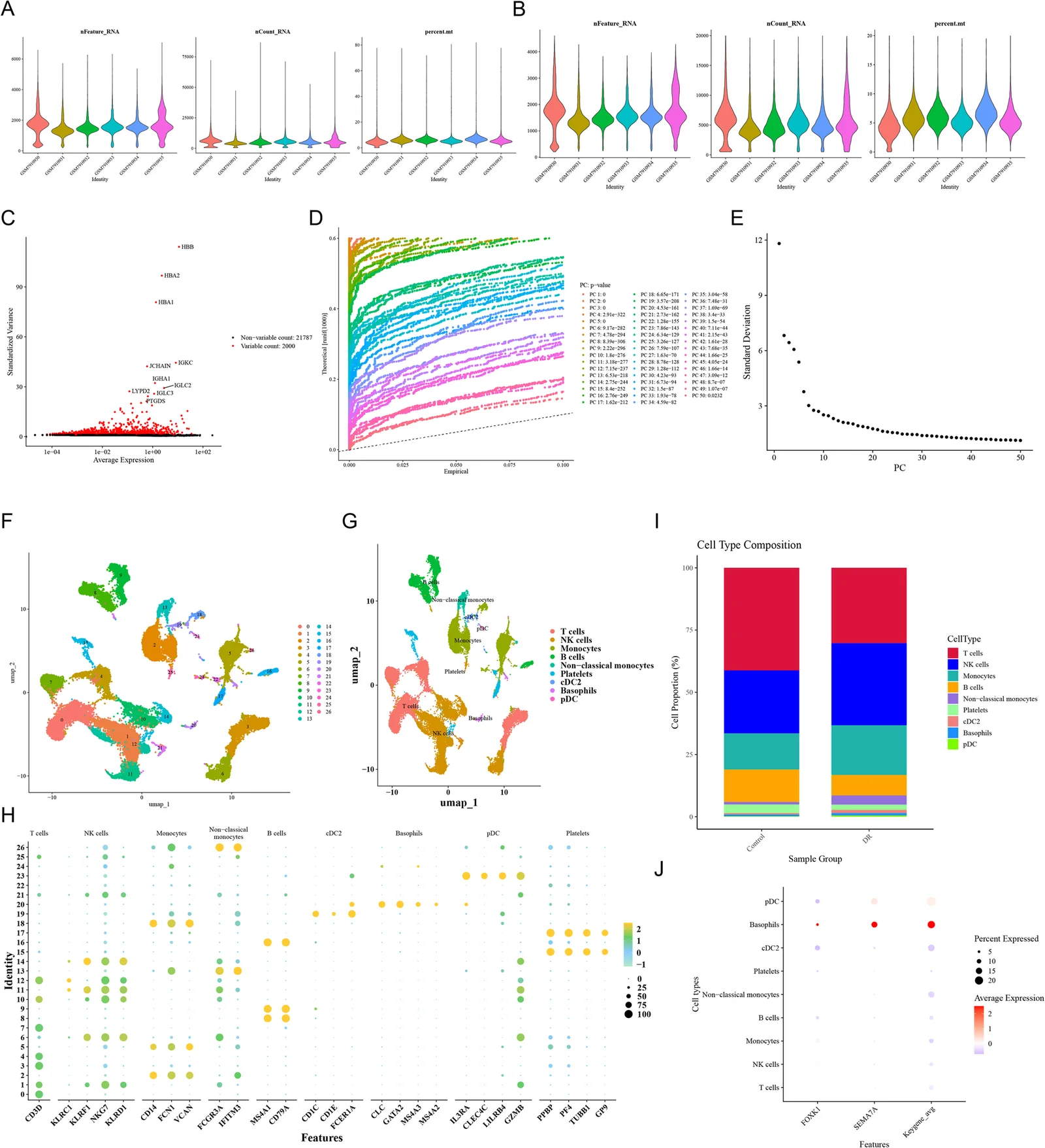
scRNA-seq analysis. **A** (Pre-QC)- **B **(Post-QC): Left: Number of genes before single-cell filtering. Middle: Total mRNA molecules. Right: Mitochondrial gene percentage. **C** Genes in red represent the top 2,000 highly variable genes, while genes in black represented non-highly-variable genes. **D** Empirical p-values (x-axis, reflecting the actual dispersion of each PC) were plotted against theoretical quantiles (y-axis), with the p-value for each PC labeled. **E** Principal component number (x) vs. standard deviation (y). **F** UMAP clustering of cell populations from all samples. **G** UMAP plot with cell type annotation. **H** Bubble plot showing the expression patterns of canonical marker genes for cell type annotation. **I** Expression profiles across cell subsets among different groups. **J** Expression of key genes across various cell populations

After preliminary screening, a total of 23,787 genes and 49,332 cells were obtained from the GSE248284 dataset (Fig. 7A, B). After normalizing and reducing the dimensionality of the data, 2,000 highly variable genes, 30 PCs, and 27 cell clusters were obtained (Fig. 7C-F). A total of 27 cell clusters were annotated into 9 cell types, including T cells, NK cells, monocytes, non-classical monocytes, B cells, type 2 conventional dendritic Cell (cDC2), basophils, platelets, plasmacytoid dendritic cell (pDC) (Fig. 7G). A bubble plot displayed high specificity of marker genes, confirming accuracy of annotation (Fig. 7H). Bar charts showed that, compared with the control group, the proportions of NK cells, basophils, and monocytes were increased, whereas those of T cells were decreased in the DR group (Fig. 7I). In terms of key gene expression, the average expression levels of FOXK1 and SEMA7A were the highest in basophils (Fig. 7J).

### Construction of the molecular regulatory network and molecular docking for predicted drugs

**Fig. 8:**
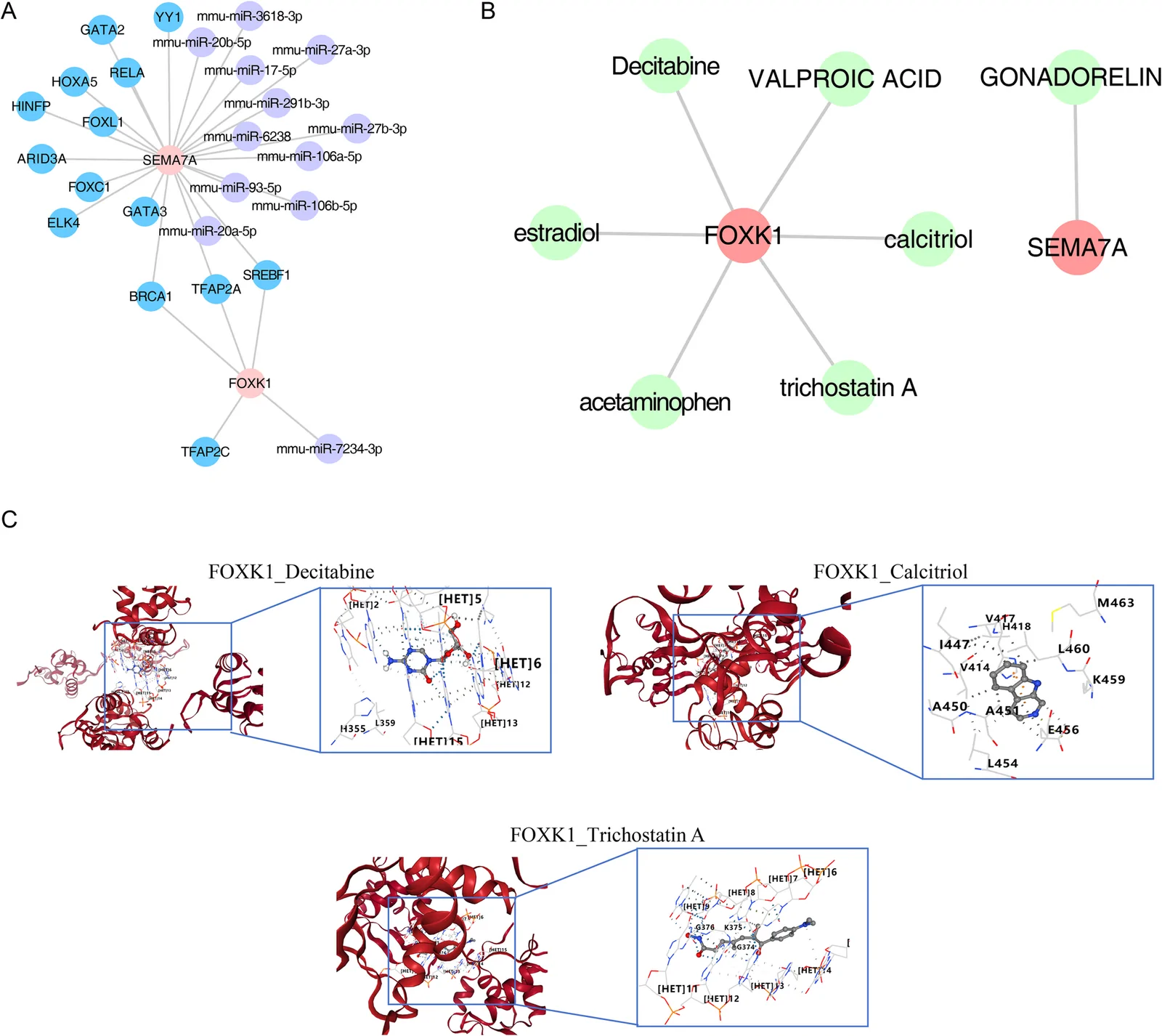
Construction of regulatory network and drug prediction analysis. **A** TF-mRNA-miRNA regulatory network for the key genes FOXK1 and SEMA7A. Red nodes represent key genes, blue nodes represent transcription factors, and purple nodes represent miRNAs. **B** Drug-key gene interaction network. Red nodes indicate key genes; green nodes indicate drugs targeting the key genes. **C** Molecular docking models visualizing the binding conformations of the top three predicted drugs (decitabine, calcitriol, trichostatin A) with the protein encoded by FOXK1

To elucidate the upstream regulatory mechanisms of key genes, a TF-mRNA-miRNA regulatory network was constructed. As a result, FOXK1 was predicted to be regulated by four TFs, while SEMA7A was targeted by 13 TFs. Additionaly, 1 miRNA for FOXK1 and 11 key miRNAs for SEMA7A were predicted. The TF-mRNA-miRNA merge network providing insights into potential transcriptional and post-transcriptional regulation of FOXK1 and SEMA7A (Fig. 8A). For example, SEMA7A and FOXK1were both targeted by BRCA1, TFAP2A and SREBF1. Meanwhile SEMA7A was targeted by mmu-miR-3618-3p, while FOXK was targeted by mmu-miR-7234-3p.

To identify potential therapeutic compounds targeting the key genes, DSigDB database was used for drug prediction, excluding carcinogens and non-drug-like compounds. FOXK1 was predicted to interact with five drugs including decitabine, estradiol acetaminophen, while SEMA7A was associated with one drug gonadorelin. (Fig. 8B). Further molecular docking analysis indicated that the binding energies of the top three drugs, decitabine, calcitriol, and trichostatin A, to FOXK1 were − 7.8, -8.6, and − 8.2 kcal/mol, respectively (Fig. 8C and Supplementary Table 15). These computational results suggest favorable binding potential between the above compounds and FOXK1 protein, which provides preliminary clues for subsequent experimental validation.

### Expression validation of key genes by RT-qPCR

**Fig. 9:**
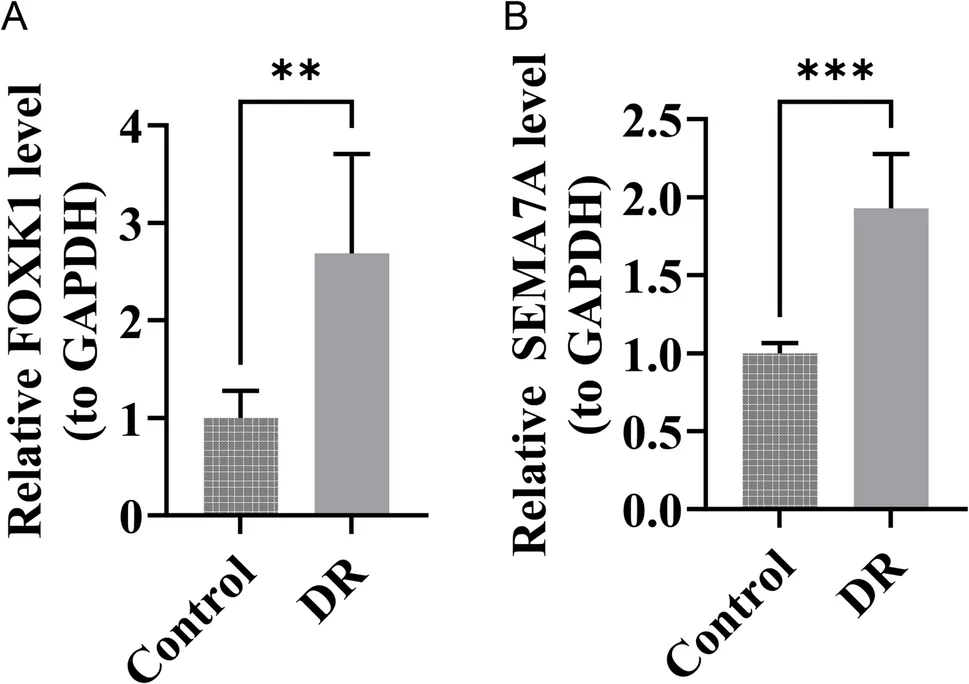
RT-qPCR anlysis. **A** Relative mRNA expression level of FOXK1 in the DR group compared to the control group. **B** Relative mRNA expression level of SEMA7A in the DR group compared to the control group. (**p < 0.01, ***p < 0.001)

The current analysis revealed marked upregulation of two critical transcripts in the diabetic retinopathy patient cohort compared to healthy control subjects. Notably, FOXK1 (Forkhead Box K1) demonstrated statistically significant elevation by 2.69-fold (fold change ≈ 2.7, 95% CI: 2.1–3.5, *p* = 0.0075), whereas SEMA7A (Semaphorin 7 A) exhibited a 1.93-fold increase in transcript abundance (fold change ≈ 1.9, 95% CI: 1.5–2.3, *p* = 0.0004). These differential expression profiles indicate the probable regulatory roles of both genetic elements in the molecular cascades governing diabetic retinopathy onset and disease progression. Higher data variability of FOXK1 in the DR group (standard deviation: 1.0233 vs. 0.278 in controls) may reflect sample heterogeneity or technical fluctuations, while SEMA7A exhibited relatively concentrated data distribution (standard deviation: 0.3487) (Fig. 9A, B and Supplementary Table 16). Notably, experimental results were highly consistent with bioinformatics analysis in gene expression trends, supporting both genes as potential biomarkers for DR.

## Discussion

DR is one of the most common microvascular complications of diabetes, potentially leading to vision loss or blindness.Diabetic retinopathy (DR) is not only a localized ocular disorder but also a systemic disease closely associated with systemic inflammation and immune dysregulation [37]. The gene expression profile of peripheral blood mononuclear cells (PBMCs) may reflect the systemic inflammatory state of DR [38]or disease-related systemic molecular alterations [39], which could be linked to retinal pathology (e.g., through immune cell infiltration [40]). This study utilized transcriptomic data obtained from the GEO database and analyzed it in conjunction with m^6^A-related and PCDRGs identified from the literature. Through differential expression, intersection, and correlation analyses, 58 candidate genes were identified. Subsequent screening revealed Forkhead box K1 (FOXK1) and Semaphorin 7 A (SEMA7A) as key genes. Functional characterization via enrichment and localization analyses elucidated their roles. A nomogram prediction model constructed based on these findings demonstrated good performance. Additionally, regulatory networks were built, and potential drugs were predicted, with some exhibiting strong binding affinity to the key gene FOXK1.

FOXK1 (Forkhead box protein K1), acting as a transcriptional regulator, plays a pivotal role in insulin signaling by directly binding to the promoters of target genes involved in processes such as glucose metabolism and mitochondrial function [41]. In the liver, insulin enhances FOXK1 binding to promoters/enhancers of nearly 75% of its target genes (e.g., LARS1, TIMM22, genes involved in glucose metabolism), thereby coordinating the transcriptional regulation of cellular cycle, autophagy, and metabolic pathways [41]. Notably, some FOXK1 target genes are directly implicated in autophagy (e.g., EZH2, FOXK1 itself), and dysregulation of autophagy has been demonstrated to contribute to fibroblast differentiation in pulmonary fibrosis [42]– a mechanism potentially analogous to hyperglycemia-induced retinal cell damage in DR. Although direct evidence linking FOXK1 to retinopathy is currently lacking, its central position within the metabolism-autophagy axis, particularly in regulating insulin sensitivity [41, 42], suggests its potential involvement in influencing DR progression through the modulation of m^6^A modification-associated PCD pathways (e.g., autophagic cell death).Notably, existing reports have indicated that m^6^A modification can regulate the expression of FOXK family genes in metabolic diseases by targeting YTHDF2-mediated mRNA degradation [43], which supports the potential existence of a similar regulatory mode in DR. Furthermore, the transcriptional activity of FOXK1 can be synergistically enhanced by the viral protein ORF45 [44], indirectly supporting the plasticity of its transcriptional regulatory network. In DR, FOXK1 function may become dysregulated due to metabolic stress, positioning it as a potential biomarker for early diagnosis.More importantly, FOXK1 has been confirmed to directly regulate a series of genes related to the cell cycle and cell survival. For example, in cancer research, FOXK1 promotes cell survival by inhibiting the expression of pro-apoptotic genes [45]. In the context of DR, metabolic stresses such as high glucose may lead to FOXK1 dysfunction, causing it to lose normal regulatory control over its downstream PCD-related target genes. This disrupts the balance between cell survival and death, driving the abnormal death of retinal cells.

SEMA7A (Semaphorin-7 A) plays a pivotal role in integrin-mediated signaling pathways, critically regulating processes such as cell adhesion and migration, thereby impacting diverse pathological states [46, 47]. SEMA7A drives epithelial-mesenchymal transition (EMT) in retinal pigment epithelial (RPE) cells by promoting focal adhesion complex assembly and activating PTK2/FAK1 kinase, which in turn phosphorylates the downstream effectors MAPK1 and MAPK3 [47]. Experimental studies demonstrate that SEMA7A overexpression directly induces EMT in RPE cells within a proliferative vitreoretinopathy (PVR) model. A hallmark of this process is the loss of epithelial marker expression and a concomitant gain in mesenchymal marker expression. Conversely, SEMA7A silencing significantly suppresses TGF-β1-induced EMT progression and attenuates activation of the PI3K-AKT/MAPK signaling pathway [47].

Furthermore, SEMA7A exerts significant immunomodulatory functions. For instance, in the nervous system, alterations in SEMA7A levels are associated with processes like axon guidance and cell adhesion, potentially impacting inflammatory responses and brain connectivity integrity [48]. Within the tumor microenvironment, SEMA7A mediates direct interactions between tumor cells and fibroblasts, leading to upregulation of IGFBP-3 and IL-17RB. This subsequently enhances cellular migration and invasive capabilities, highlighting its promigratory role in malignancies such as pancreatic cancer [46]. The potential of SEMA7A as a therapeutic target is strongly supported by these findings, which lay a solid groundwork for future investigation [46–48]The association between SEMA7A and programmed cell death (PCD) primarily lies in its potent pro-inflammatory signaling capacity. SEMA7A activates downstream FAK and MAPK pathways via integrins [49]. These pathways not only drive epithelial-mesenchymal transition (EMT) but also serve as key regulators of inflammatory cell death, such as pyroptosis [50, 51]. More directly, in a model of metabolic dysfunction-associated fatty liver disease, an activated variant of SEMA7A has been shown to enhance binding to integrin β1, triggering the PI3K/Akt pathway, inducing reactive oxygen species (ROS) accumulation, thereby activating the NLRP3 inflammasome and promoting IL-1β maturation, ultimately leading to hepatocyte pyroptosis [52]. Notably, the microenvironment of high glucose-induced diabetic retinopathy (DR) is also characterized by oxidative stress, NLRP3 inflammasome activation, and elevated IL-1β levels [53, 54]. Therefore, we propose a plausible hypothesis: in the chronic inflammatory and metabolic stress environment of DR, SEMA7A may activate the NLRP3 inflammasome-mediated pyroptosis pathway through a similar integrin–FAK/MAPK/PI3K-Akt–ROS signaling axis, thereby exacerbating inflammatory injury in retinal vascular endothelial cells and neuroglial cells.

In GSEA analysis, FOXK1 and SEMA7A demonstrated significant enrichment in pathways such as “Ribosome,” “Spliceosome,” and “Parkinson’s disease,” revealing their potential roles in retinal pathology. The ribosome is the macromolecular complex responsible for mRNA translation during protein synthesis [55]. Ribosome biogenesis involves multiple coordinated steps, including rRNA transcription, rRNA splicing, rRNA modification, ribosomal assembly, and nucleocytoplasmic export of ribosomal precursors [55, 56]. Impairment of ribosomal integrity or dysregulation of protein biosynthesis may contribute to human diseases such as diabetic retinopathy (DR) and other ocular disorders [57]. Evidence indicates that ribosome-related pathways influence DR pathogenesis through the modulation of cellular signaling cascades and retinal development. Specifically, the ribosomal protein RPS4Y1 is upregulated under hyperglycemic conditions and promotes endothelial cell apoptosis and inflammation via p38 MAPK signaling activation, potentially contributing to vascular dysfunction in DR [58]. Furthermore, mutations in ribosome biogenesis factors such as kri1l, a core component of the SSU processome, cause defective rRNA processing that leads to retinal developmental abnormalities. This manifests as cell cycle arrest and apoptosis induction, mechanisms relevant to DR-associated ocular pathology [57]. The enrichment of FOXK1 and SEMA7A in the ribosome pathway indicates a statistical association between these genes and biological processes involving protein synthesis. However, this does not establish a direct regulatory relationship between these genes and ribosome biogenesis, nor does it prove their involvement in m⁶A-dependent processes or PCD-related pathways. We hypothesize that FOXK1 and SEMA7A may participate in the regulation of protein synthesis and apoptosis-related energy metabolism in DR. This hypothesis requires confirmation through direct experimental validation.

Considerable heterogeneity was observed in the immunoinfiltration landscape, with 12 immune cell subtypes exhibiting substantial differences between DR patients and control subjects.Key genes FOXK1 and SEMA7A show significant correlations with specific immune subsets, such as natural killer cells. This observation aligns with the pathological mechanisms observed in various ophthalmic diseases: Müller glial cells, critical immunomodulatory cells in the retina, contribute to disrupted retinal homeostasis and tissue injury when functionally impaired under pathological conditions. This mechanism has been linked to dysregulation of the immune microenvironment in neurodegenerative eye diseases [59]. Recent studies reveal that HDAC6-mediated activation of the NLRP3 inflammasome represents a core pathogenic mechanism in DR. It directly drives retinal inflammation and immune cell activation, leading to vascular leakage and neuronal damage. Notably, genetic deletion or specific inhibition of HDAC6 significantly suppresses inflammasome activation. Furthermore, studies involving bone marrow transplantation experiments have established that HDAC6 signaling in immune cells plays a crucial regulatory role in the pathological progression of DR [60]. Notably, our immune infiltration analysis was performed based on whole-blood transcriptome data, which reflect systemic immune alterations rather than the intraocular immune microenvironment. Although the peripheral blood immune profile may be associated with retinal pathological changes through systemic inflammatory mechanisms, it cannot directly represent cell-specific immune responses within the retina. Therefore, our findings regarding immune cell dysregulation should be interpreted as manifestations of DR-associated systemic immune alterations. Further studies utilizing retinal tissues or animal models are warranted in the future to verify whether similar immune cell infiltration patterns also exist in the retina. Furthermore, in this study, a human single-cell transcriptomic dataset (GSE248284) of DR was utilized to systematically delineate the immune cell atlas within the lesional microenvironment. After quality control, dimensionality reduction, clustering and cell type annotation, all cells were finally classified into nine immune cell subsets. The cellular proportion analysis revealed that the proportions of NK cells, monocytes and basophils were significantly elevated in samples from patients with DR relative to healthy controls, whereas the percentage of T cells decreased markedly. These findings indicated that disorders in both adaptive and innate immunity jointly contributed to the initiation and progression of DR, and chronic low-grade inflammatory infiltration represented a typical hallmark of the local microenvironment in DR [61, 62]. Notably, both FOXK1 and SEMA7A exhibited the highest average expression levels in basophils. Although basophils have rarely been investigated in the context of DR, they might participate in immune regulation and alterations in vascular permeability via releasing mediators such as histamine and interleukin-4 (IL-4), thereby closely associating with the chronic inflammatory status of DR [63, 64]. Our results suggested that FOXK1 and SEMA7A might be involved in the immunopathological processes of DR by regulating basophil functions. This single-cell analysis defined the cell-type-specific expression patterns of the two core genes, supplemented molecular evidence regarding the regulation of immune imbalance in DR by m⁶A modification- and programmed cell death-related genes at the cellular subset level, and provided a novel research direction for subsequent interventions targeting basophils to alleviate inflammatory injury in DR.

The consistency between RT-qPCR results and gene expression profiles confirms the reliability of FOXK1 and SEMA7A in the diagnosis of DR: both promote DR progression by regulating metabolic-autophagy imbalance (FOXK1), epithelial-mesenchymal transition (EMT)-mediated fibrosis (SEMA7A), and neuro-immune axis dysregulation, while m^6^A modification may act as an upstream regulatory factor in these processes.Future research could focus on targeting the HDAC6/SEMA7A signaling or intervening in the FOXK1-autophagy pathway to develop novel immunotherapeutic strategies for DR.

Previous experimental studies have demonstrated that FOXK1 regulates apoptosis, migration and angiogenesis in high glucose-induced vascular endothelial cells through the p-AKT/AKT signaling pathway [65], providing functional evidence supporting our bioinformatics findings. SEMA7A has been identified as a hub molecule associated with proliferative diabetic retinopathy (PDR) [66]. However, neither of these studies addressed the regulatory relationship between m^6^A modification and programmed cell death (PCD). This study is the first to incorporate both into an m^6^A-PCD regulatory framework, demonstrating that FOXK1 and SEMA7A synergistically regulate the pathological progression of DR through pathways such as ribosome biogenesis and oxidative phosphorylation. Additionally, a TF-mRNA-miRNA regulatory network comprising 14 transcription factors and 12 miRNAs (e.g., BRCA1-FOXK1-mmu-miR-7234-3p) was constructed. The study clarified the negative correlation between FOXK1 and natural killer cells (cor = -0.65, *p* < 9.54 × 10⁻¹⁶) and validated the high-affinity binding of drugs such as decitabine to FOXK1 through molecular docking (binding energy: -7.8 kcal/mol). Notably, the above compounds are not conventional drugs for diabetic retinopathy treatment. All findings in this section are obtained from database mining and molecular docking simulations, and relevant pharmacological effects and target binding effects remain to be verified by cell-based functional assays. This research is the first to reveal the central role of FOXK1 and SEMA7A in m^6^A -associated PCD, addressing the gaps in regulatory network completeness and mechanistic depth in previous studies, and providing a new theoretical basis for precise targeted therapy of DR.

The investigation revealed FOXK1 and SEMA7A as central regulators in the network of m^6^A-dependent programmed cell death associated with diabetic retinopathy. Predicted drugs, such as Decitabine, bind well to FOXK1, indicating their potential as therapeutic candidates. However, this study has certain limitations. First, this study divided sample groups based on the ssGSEA scores of 27 m⁶A regulatory genes, assuming that their expression changes could reflect the overall m⁶A modification level. Therefore, the m⁶A-related differentially expressed genes screened by this grouping approach carry certain methodological biases. Subsequent studies can optimize the grouping strategy by integrating global m⁶A dot blot assays or LC-MS quantitative experiments to improve screening reliability. Second, this study cannot yet confirm whether FOXK1 and SEMA7A are directly regulated by m⁶A at the post-transcriptional level. Future epitranscriptomic experiments, including m⁶A sequencing and MeRIP-qPCR, will be performed to verify whether authentic m⁶A modification sites exist on the mRNA transcripts of these two genes. Third, the clinical peripheral blood samples used for RT-qPCR validation in this study only included 5 DR cases and 5 controls. The relatively small sample size may reduce the statistical power, and there are certain limitations in extrapolating the experimental conclusions to a broader population. Meanwhile, restricted by the clinical specimen storage conditions, the collection of some clinical indicators was limited, making it difficult to completely avoid potential confounding factors. Subsequent studies will continuously enroll more patient samples for expression validation in larger cohorts and simultaneously improve the comprehensive collection of clinical information to further enhance the reliability of the results.

## Conclusion

This study focused on DR. Transcriptomic data was obtained from the GEO database and analyzed in conjunction with m^6^A-related and PCDRGs identified in the literature. Through differential expression, intersection, and correlation analysis, 58 candidate genes were identified. Further screening revealed FOXK1 and SEMA7A as key genes. Enrichment analysis and localization analysis clarified their functional characteristics. Derivation of a nomogram from these findings yielded a model with satisfactory performance upon validation. Additionally, regulatory networks were constructed, and potential drugs were predicted. The analysis indicated strong binding affinity between part of the predicted drugs and the key gene FOXK1. This research provides new insights for DR study and treatment.

## Supplementary Information

Supplementary Material 1: The list of N6-methyladenosine modification (m6A)-related genes.

Supplementary Material 2: The list of PCD-related genes.

Supplementary Material 3: Baseline clinical characteristics of the participants.

Supplementary Material 4: The experimental methods and primer sequences for RT-qPCR.

Supplementary Material 5: Differentially expressed genes (DRDEGs) between DR samples and control samples in the training dataset.

Supplementary Material 6: m6A-related differentially expressed genes (m6ADEGs) between DR samples and control samples.

Supplementary Material 7: Candidate genes identified by Spearman correlation analysis between 85 DRPCDs genes and 481 m6ADRGs.

Supplementary Material 8: Sensitivity analysis of gene screening under different correlation coefficient thresholds.

Supplementary Material 9: GO enrichment analysis.

Supplementary Material 10: KEGG enrichment analysis.

Supplementary Material 11: Significantly enriched diseases associated with the candidate genes.

Supplementary Material 12: GSEA enrichment analysis of FOXK1.

Supplementary Material 13: GSEA enrichment analysis of SEMA7A.

Supplementary Material 14: Correlation results between SEMA7A and differentially infiltrated immune cells.

Supplementary Material 15: The binding energy between key gene and drugs.

Supplementary Material 16: Differential expression of FOXK1 and SEMA7A between DR patients and healthy controls.

## Data Availability

The datasets analysed during the current study are available in the [Gene Expression Omnibus] repository [ https://www.ncbi.nlm.nih.gov/geo/ ].
