# Supplementary material for: Identification of FOXK1 and SEMA7A as key genes associated with m6A-related programmed cell death in diabetic retinopathy
Source: Hereditas. 2026 Jul 10;163:104. doi: 10.1186/s41065-026-00710-w (PMC13640323; doi:10.1186/s41065-026-00710-w)
Supplement: Supplementary file 1 [file 41065_2026_710_MOESM4_ESM.docx]

**Supplementary Table 4: The experimental methods and primer sequences for RT-qPCR**

Results of concentration measurement

| Number | Group | concentration(ng/uL) | A260/A280 | A260/A230 |
| --- | --- | --- | --- | --- |
| 1 | Control | 160.68 | 1.605 | 0.312 |
| 2 | Control | 189.36 | 1.352 | 0.258 |
| 3 | Control | 176.4 | 1.379 | 0.363 |
| 4 | Control | 153.04 | 1.323 | 0.228 |
| 5 | Control | 132.12 | 1.396 | 0.232 |
| 6 | DR | 206.2 | 1.325 | 0.238 |
| 7 | DR | 152.8 | 1.237 | 0.203 |
| 8 | DR | 123.6 | 1.24 | 0.229 |
| 9 | DR | 113.02 | 1.345 | 0.281 |
| 10 | DR | 120.12 | 1.521 | 0.312 |

Reverse transcription of mRNA was conducted using the Hifair^®^ Ⅲ 1st Strand cDNA Synthesis SuperMix for qPCR Kit from Yeasen Biotech. Briefly, all components of the reverse transcription kit were taken out, allowed to thaw at room temperature, briefly centrifuged, and placed on ice. Subsequently, the reagents and solutions were added in sequence on ice according to the specified protocol.

Composition of the reverse transcription reaction system（Component vs. Volume）

| **Component** | **Volume** |
| --- | --- |
| 5×gDNA digester Mix | 3ul |
| 4×Hifair® Ⅲ SuperMix plus | 5ul |
| Total RNA | 2ug |
| Nuclease-Free Water | Add to 20ul |

After brief centrifugation, reverse transcription was performed on a conventional PCR machine under the following conditions. Composition of the qPCR reaction system (Component vs. Volume)

| **Temperature** | **Time** |
| --- | --- |
| 25℃ | 5min |
| 55℃ | 15min |
| 85℃ | 5min |
| 4℃ | hold |

First, dilute the above reverse transcription product cDNA 5-20 fold with ddH₂O (RNase/DNase free). Then set up the qPCR reaction according to the following reaction system. Reverse transcription program (Temperature vs. Time)

| **Component** | **Volume** |
| --- | --- |
| cDNA | 3ul |
| 2xUniversal Blue SYBR Green qPCR Master Mix | 5ul |
| Forward primer (10µM) | 1ul |
| Reverse primer (10µM) | 1ul |

During sample loading, check for air bubbles in the pipette tip each time after aspirating the liquid. After dispensing into the well, inspect the tip for any residual liquid; if present, expel it completely into the same well. Perform pipetting operations smoothly without interruptions for unrelated tasks to ensure consistency across replicate wells.

Following a brief centrifugation, conduct 40 cycles of qPCR on a CFX96 Real-Time PCR Detection System under the conditions specified below. Generate amplification curves and melting curves, and record the Ct values.

Amplification Conditions (Temperature vs. Time)

|  | **Temperature** | **Time** |
| --- | --- | --- |
| Pre-denaturation | 95℃ | 1min |
| Denaturation | 95℃ | 20s |
| Annealing | 55℃ | 20s |
| Extension | 72℃ | 30s |

The relevant primer sequences are as follows:

| **Primer** | **Sequence** | |
| --- | --- | --- |
| FOXK1 F | CAGGGAGAAGCCGTGAATGT | |
| FOXK1 R | GTGACTTGGAGACCGCTTCA |  |
| SEMA7A F | TTCAGCCCGGACGAGAACT |  |
| SEMA7A R | GAACCGAGGGATCTTCCCAT |  |
| Reference Gene-GAPDH F | ATGGGCAGCCGTTAGGAAAG |  |
| Reference Gene-GAPDH R | AGGAAAAGCATCACCCGGAG |  |
